# Successful tracheal regeneration using biofabricated autologous analogues without artificial supports

**DOI:** 10.1038/s41598-022-24798-y

**Published:** 2022-11-24

**Authors:** Shohei Hiwatashi, Ryosuke Iwai, Yasuhide Nakayama, Takeshi Moriwaki, Hiroomi Okuyama

**Affiliations:** 1 Department of Pediatric Surgery, Osaka University Graduate School of Medicine, Osaka, 565-0871 Japan; 2 Institute of Frontier Science and Technology, Okayama University of Science, Okayama, 700-0005 Japan; 3 Osaka Laboratory, Biotube Co., Ltd, Osaka, 565-0842 Japan; 4 Faculty of Science and Technology, Hirosaki University, Aomori, 036-8561 Japan

**Keywords:** Biotechnology, Tissue engineering, Paediatric research

## Abstract

Tracheas have a tubular structure consisting of cartilage rings continuously joined by a connective tissue membrane comprising a capillary network for tissue survival. Several tissue engineering efforts have been devoted to the design of scaffolds to produce complex structures. In this study, we successfully fabricated an artificial materials-free autologous tracheal analogue with engraftment ability by combining in vitro cell self-aggregation technique and in-body tissue architecture. The cartilage rings prepared by aggregating chondrocytes on designated culture grooves that induce cell self-aggregation were alternately connected to the connective tissues to form tubular tracheal analogues by subcutaneous embedding as in-body tissue architecture. The tracheal analogues allogeneically implanted into the rat trachea matured into native-like tracheal tissue by covering of luminal surfaces by the ciliated epithelium with mucus-producing goblet cells within eight months after implantation, while maintaining their structural integrity. Such autologous tracheal analogues would provide a foundation for further clinical research on the application of tissue-engineered tracheas to ensure their long-term functionality.

## Introduction

An ideal implant that can replace and regenerate a tissue is expected to have a tissue-specific structure pre-incorporated into the cells, extracellular matrices, and capillary networks. In tissue engineering, biodegradable polymers have been synthesized to fabricate tubular structures like blood vessels, and more complex structures like ears^1–3^. For example, Roh et al. developed tissue-engineered vascular grafts by seeding bone marrow-derived mononuclear cells into biodegradable tubes made of polylactic acid. The grafts can replace vascular tissues through the infiltration of endothelial and smooth muscle cells from native blood vessels after implantation^4^.

There have been attempts to develop artificial tracheas using biodegradable polymers. The trachea, which has horseshoe-shaped cartilage rings wrapped around the conduit tissue, has the strength and flexibility to withstand respiratory pressure depending on its specific structure. Therefore, most tissue-engineered tracheas (TETs) are designed to mimic the morphology of native trachea, where chondrocytes are seeded in biodegradable polymer tubes^5,6^. However, in most TETs, granulation tissues are formed on the lumen surface by foreign body reactions, inflammatory reactions, and infections on biodegradable scaffold polymers, resulting in only a few survivors three months after the TET implantation^5,7^. In addition, ciliated epithelial cells rarely cover the lumen surface owing to the granule formation. Eventually, the original cartilage tissue deteriorates and is replaced with a granulation tissue because of insufficient capillary networks needed to supply oxygen and nutrients^5^. Therefore, the implantable artificial trachea should be a "living" trachea-like tissue with a cartilage-like shape and capillary network without artificial materials.

We have developed a one-day cell self-aggregation technique (CAT) using a synthesized zwitterionic polymer^8–10^. The cells seeded on the polymer-coated surface adhered within a few hours to form a gapless monolayer cell sheet. After several hours of incubation, the sheet started to peel off the surface from the outer edge. Finally, all seeded cells aggregated to form a single cell mass after approximately one day^10^. By controlling the area of the polymer coating, we successfully formed cell aggregates with the desired 3D shape, including rings^11^.

In a separate study, we developed an in vivo tissue engineering technique called the in-body tissue architecture (iBTA). We prepared iBTA-induced tissues of various shapes and sizes, such as a tubular tissue called Biotube for vascular grafts^12,13^, sheet-like tissue called Biosheet for cornea^14^, aortic valve leaflets^15^, myocardium^16^, and others, and a more complex heart valve-like tissue called Biovalve^17^. The Biotube has already been clinically applied as an alternative blood vessel for bypass to a chronic limb-threating ischemia patient^13^ or hemodialysis patients. The patch implantation of the Biosheets into the partial tracheal defects in beagle dogs resulted in the reconstruction of the tracheal tissue by covering the ciliated epithelium on the lumen surface and the partial infiltration of chondrocyte aggregates in the middle wall^18^. When the Biotube was implanted circumferentially into the trachea, stenosis occurred within a few days owing to its insufficient strength.

Thus, in this study, we combined CAT to create cartilage rings and iBTA to create connective tissue conduits in the body for the fabrication of a scaffold-free trachea analogue without comprising any artificial materials. Histological and mechanical analyses of the fabricated tracheal analogues were carried out. Subsequently, conduit orthotopic implantation was performed to evaluate the long-term engraftment of the scaffold-free tracheal analogues.

## Results

### Fabrication of tracheal analogue

**Figure 1:**
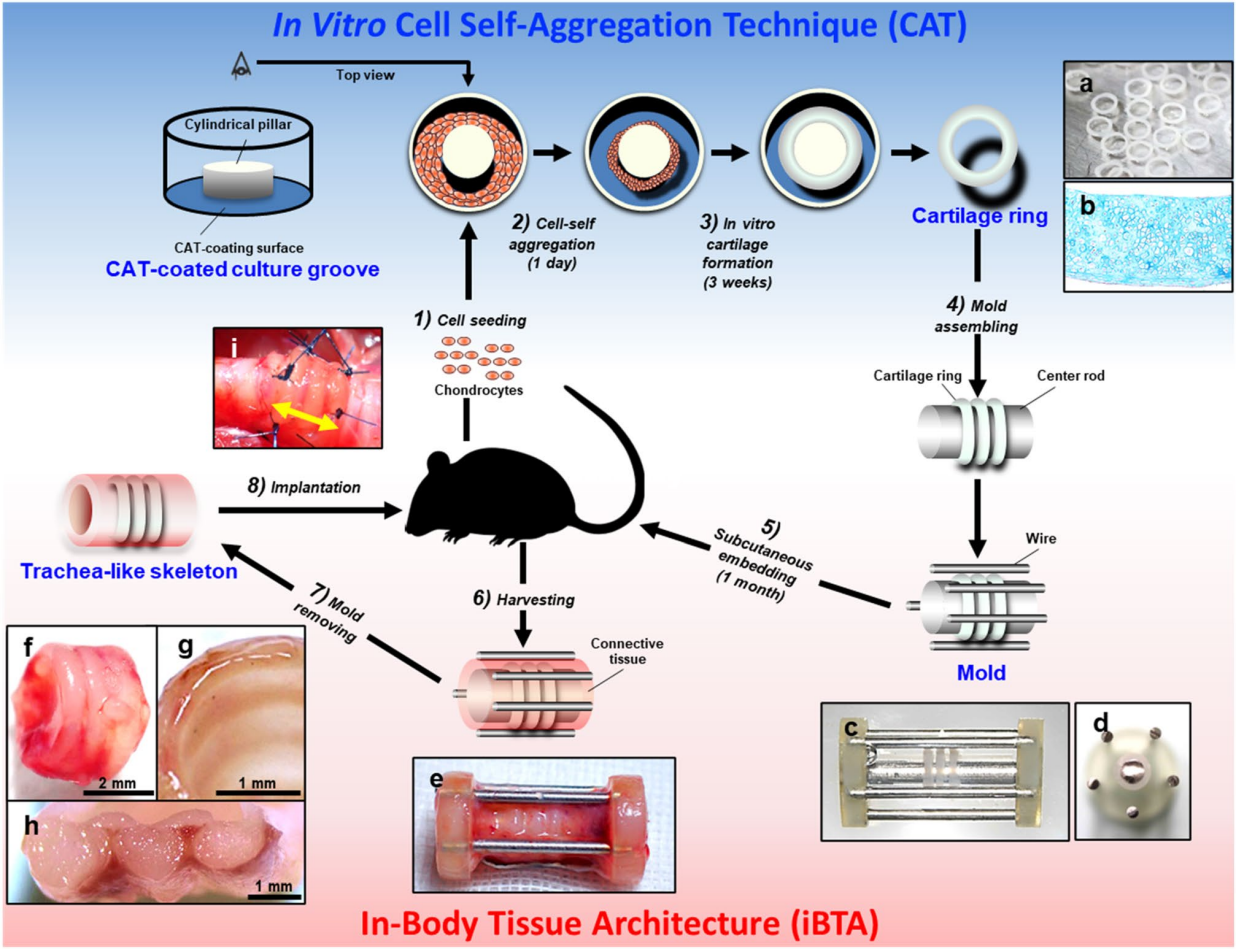
Biofabrication scheme of the tracheal analogue using in vitro cell self-aggregation and iBTA. The cartilage rings were prepared by seeding the chondrogenic lineage cells on an originally developed cell self-aggregation induction culture groove (**1**–**3**). The cartilage rings were assembled with a mold for body tissue architecture and implanted subcutaneously (**4**,**5**). After four weeks, the cartilage rings were connected to the connective tissues and capillary network, and a tracheal analogue was formed (**6**,**7**). The analogues were implanted into the trachea in an allogeneic manner (**8**). Alcian blue-stained matured cartilage rings were reproduced (**a**,**b**). Macroscopic images of the mold (**c**, side view; **d**, cross view) assembled with the cartilage rings (**e**). Macroscopic images of the outer surface (**f**), lumen (**g**), and cross-section (**h**) of the tracheal analogue formed surrounding the mold after subcutaneous implantation (**e**). Implantation view of the tracheal analogue (yellow arrow) into the trachea (**i**).

**Figure 2:**
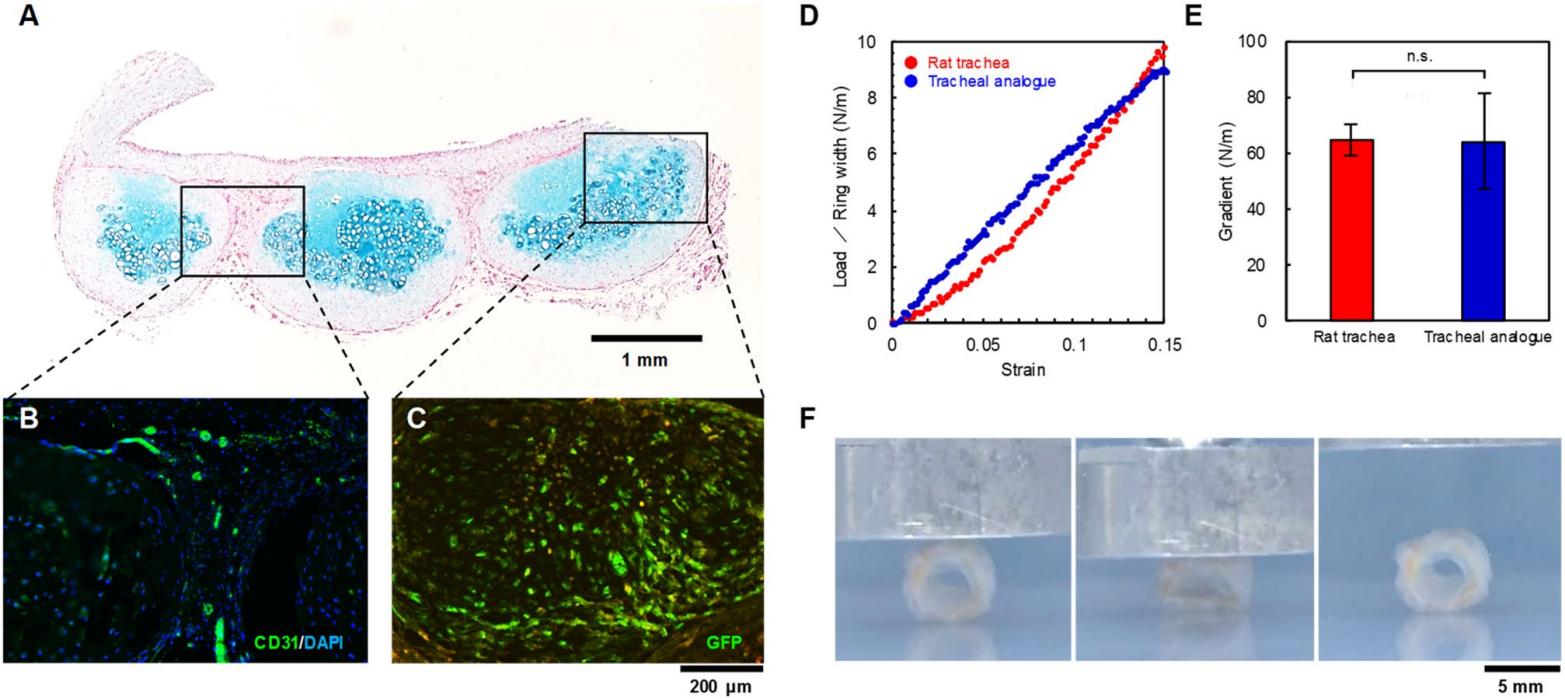
Histological analysis and mechanical properties of the tracheal analogues. The long-axis sections of the analogues stained with alcian blue dye for the cartilage proteoglycans (**A**), CD31 antibody for the capillary vessels and DAPI for the nucleus (**B**), and GFP for cell tracing (**C**). Typical examples of deformation curves of the tracheal analogue and rat native trachea (**D**). The gradients of load-strain curves in 0.05 ~ 0.1 strain range for the tracheal analogue (*n* = 5) or rat native trachea (*n* = 3) (**E**). Sequential photographs of the deformation and repair of tracheal analogue (**F**).

**Figure 3:**
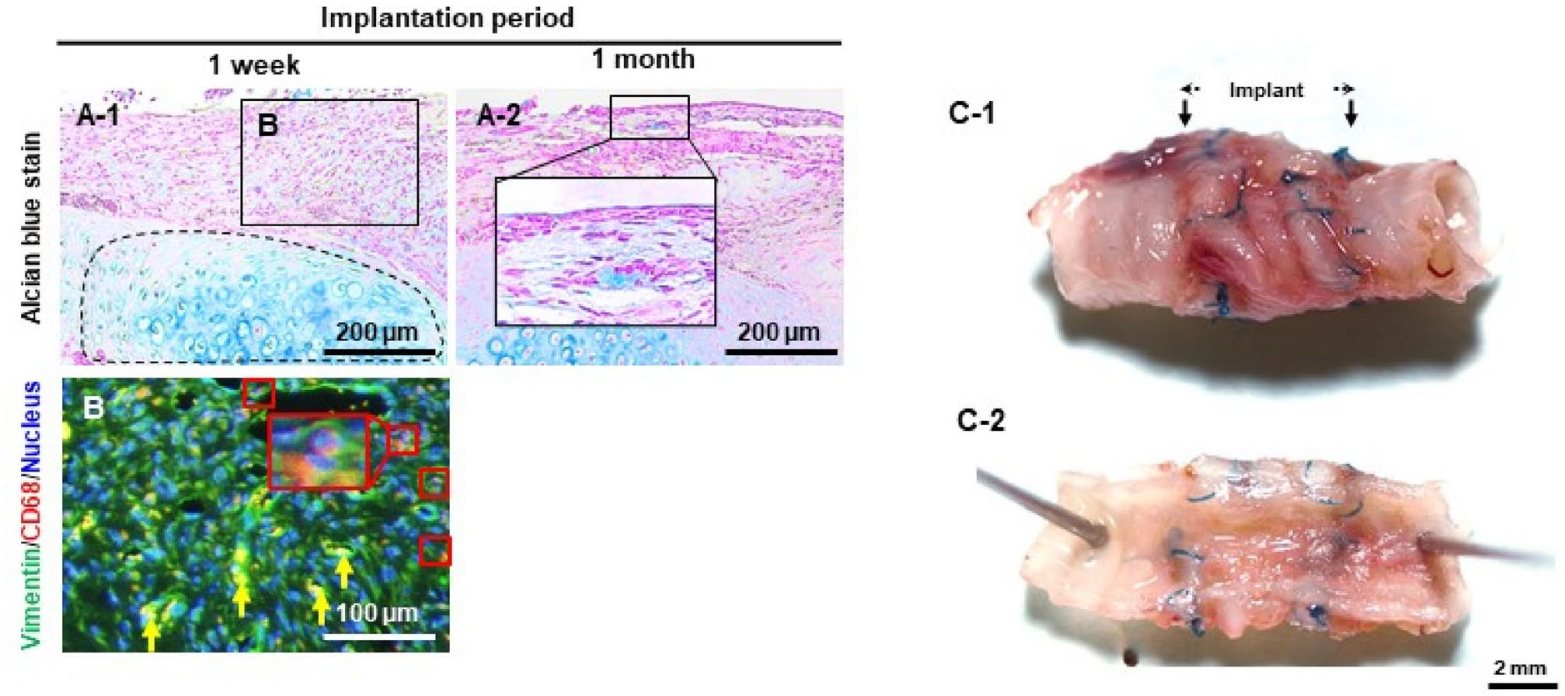
Histological analysis of tracheal analogue implants in the subacute phase. The sections of tracheal analogues implanted after 1 week (**A-1** and **B**) and 1 month (**A-2**) stained with alcian blue dye (**A-1** and **A-2**) and vimentin and CD68 antibody (**B**). Macroscopic view of the tracheal analogue after implanting for one month at outer surface (**C-1**) and lumen (**C-2**). The dashed circles in (**A-1**) indicate the cartilage rings. The yellow arrows and red flames in B indicate erythrocytes in capillaries, identified by yellow fluorescence arising from overlapping of red and green autofluorescence, and CD68-positive cells, respectively.

Figure 1 shows the flowchart for the preparation of the tracheal analogue. The CAT-coated plates were prepared by coating specially designed zwitterionic polymers on the ring-shaped bottom of a 48-well plate. When rat chondrocytes were seeded to fill the CAT-coated surface of the plate, a monolayer was formed without gaps within approximately 1 h (Fig. 1-1). During the subsequent overnight culture, the cell monolayer was delaminated and self-aggregated into a ring shape around the cylindrical pillar (Fig. 1-2) After culturing for three weeks, the aggregates grew to a thickness of approximately 1 mm by producing a cartilage substrate (Fig. 1-3). The removal of the aggregates from the pillar yielded a mature cartilage ring with an inner diameter of 3 mm (Fig. 1a,b). The resulting cartilage ring could be easily manipulated using tweezers.

**Figure 4:**
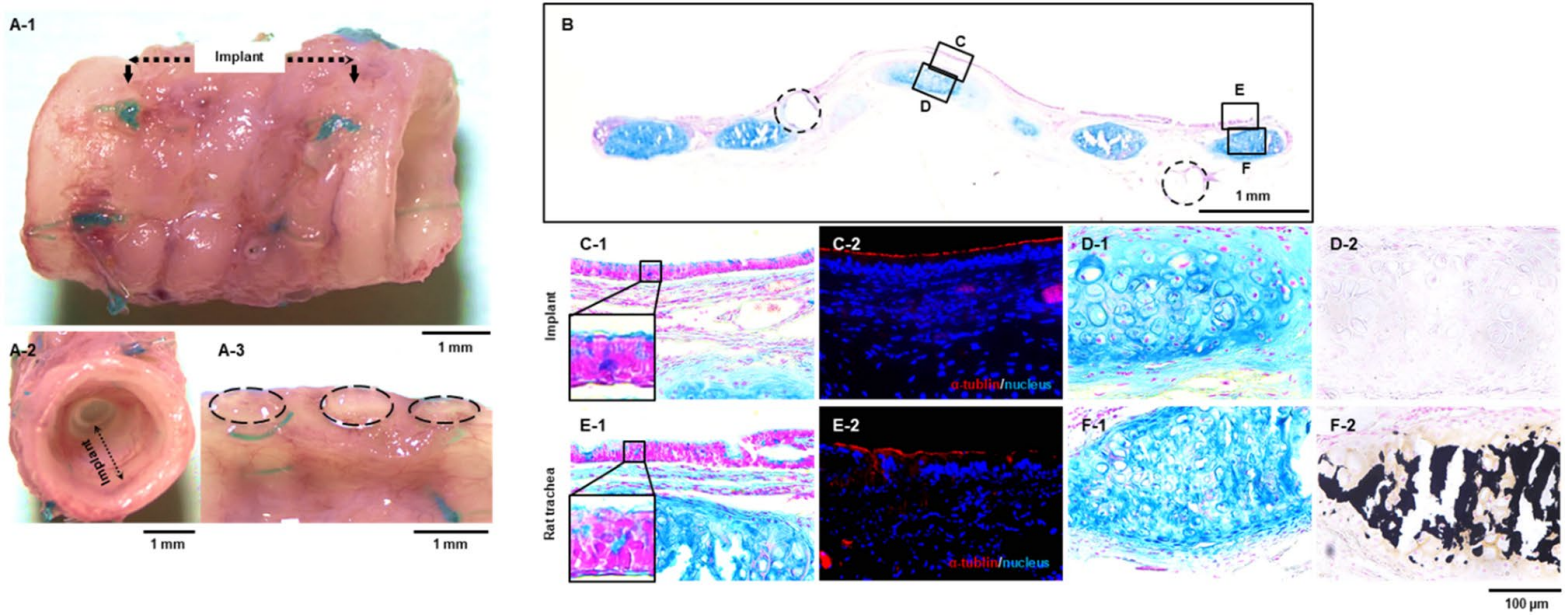
Histological analysis of tracheal analogue implant in the chronic phase. Macroscopic view of tracheal analogue 8 months after implantation at the outer surface (**A-1**), lumen (**A-2**) and longitudinal cross-section (**A-3**). Longitudinal cross-section of the tracheal analogue implanted at rat trachea for 8 months and stained in alcian blue (**B**). Magnified sections of the lumen (**C**) and cartilage (**D**) of the tracheal analogue, and native rat tracheal lumen (**E**) and cartilage (**F**) (**C-1**, **D-1**, **E-1** and **F-1**, alcian blue stain for the mucus and cartilage matirix; (**C-2**) and (**E-2**), α-tubulin for the ciliated epithelium and DAPI for the nucleus, (**D-2**) and (**F-2**); vonkossa stain for calcium deposition). The dashed circles in B indicate the anastomosis sites.

To prepare the mold for the iBTA, multiple cartilage rings were first inserted into the centre rod (Fig. 1-4). Subsequently, the outer circumference of the rod was surrounded by wires, and both ends were fastened with plastic disks to complete the mold (Fig. 1c,d). The assembled mold was subcutaneously embedded in the rats (Fig. 1-5). After four weeks of in-body incubation (Fig. 1-6), the interior of the mold was filled with red connective tissue containing several capillaries (Fig. 1e). After removing the mold (Fig. 1-7), an integrated tracheal analogue was obtained by connecting the cartilage rings with the connective tissues (Fig. 1f). The tracheal analogue had a smooth luminal surface (Fig. 1g). The observation of the cross-section revealed that the cartilage was separated and surrounded by connective tissues (Fig. 1h).

### Properties of tracheal analogue

For histological observations of the tracheal analogue, the cartilage ring was stained with alcian blue dye, indicating that the cartilage matrix of chondroitin sulfate proteoglycan was maintained (Fig. 2A). In addition, CD31-positive capillaries were found in the connecting tissue of the cartilage ring (Fig. 2B). Furthermore, most of the cells that make up the cartilage ring were expressed by the GFP, indicating that the cartilage ring was derived from cultured chondrocytes (Fig. 2C).

To investigate the ring resistance to deformation, compression testing was carried out using a universal tester. The native trachea displays a J-shaped curve (Fig. 2D). The gradients in 0.05–0.1 strain range are found similar between them (Fig. 2E). Furthermore, the analogues could restore their original shape even when deformed by at least 30% of their diameter (Fig. 2F).

### In vivo performance of tracheal analogue

The tracheal analogues with three cartilage rings having internal diameter of 3 mm and length of 3–3.5 mm were implanted into the trachea of allogenic Lewis rats (Fig. 1-8). The analogues were interposed into the native tracheas as a conduit (Fig. 1i). A total of 19 patients underwent implantation. 16 of 19 rats died before reaching the experimentally designed endpoints. Among the deaths, 11 rats died by the day after implantation. The reason for the deaths was that the tracheas were obstructed by airway secretions, due to the reduced respiratory, swallowing and sputum functions after the operation. The remaining five rats died within the next two weeks, which we attribute to respiratory failure resulting from stenosis caused by airway secretions and diminished feed intake. During the entire implantation period, no lumen obstruction due to excessive tissue growth or tissue degradation was observed. Thus, three rats surviving 1 week, 1 month, and 8 months were harvested and analysed.

One week after the implantation, there were no inflammatory cells such as granulocytes or lymphocytes as forming cluster arising from immune reactions or infections in the connective tissue membrane constituting the tracheal analogue lumen. The tissue components in the connective tissue membrane were identified as a mostly vimentin-positive mesenchymal cells originally present in tracheal analogue, blood vessels flowing erythrocytes, and small number of macrophages. In addition, the cartilage rings maintained the histologically cartilage-specific lacunar structure with alcian blue-stained images similar to those of the pre-implant tracheal analogue shown in Fig. 2A (Fig. 3A-1, within the dashed frame). At 1 month after implantation, inflammatory adhesion with the surrounding tissues and degradation of the implant was not observed (Fig. 3C-1). Moreover, the implant did not form granulation and remained smooth in the lumen (Fig. 3C-2). Histological observations showed that the lumen of the implant was covered with epithelial cells while the cartilage tissue was maintained (Fig. 3A-2), indicating the reconstruction of the trachea.

The implant 8 months after implantation showed no significant deformation or deterioration on the outer surface (Fig. 4A-1). The lumen of the implant was macroscopically smooth; thus, the boundaries to the natural trachea were indistinguishable (Fig. 4A-2). From the longitudinal cross-section of the implants, white cartilage tissues were clearly visible (Fig. 4A-3). The histological observations revealed that the implant was covered with an epithelial layer along its entire length (Fig. 4B). In detail, the epithelial cell layer was composed of two types of epithelial cells similar to native tracheal epithelium with mucus-stained goblet cells (Fig. 4C-1 and E-1) and α-tubulin-positive ciliated epithelial cells (Fig. 4C-2 and E-2). In addition, the implant maintained its cartilage matrix and specific cartilage lacuna structure (Fig. 4B, D-1, and F-1) without arising cartilage calcification (Fig. 4D-2), while the significant calcification was found in native tracheal cartilage (Fig. 4F-2).

## Discussion

TETs fabricated using tracheal cells, such as chondrocytes or epithelial cells, and scaffolds, such as biodegradable polymers, have been actively studied; however, there are limited studies that have reached the clinical stage. Therefore, even in small animals, such as rats and rabbits, most biofabricated tracheas are difficult to survive at least three months after their circumferential implantation^5–7^. In contrast, decellularized tissues might offer new therapeutic options in regenerative medicine in trachea as well. A report suggests that rabbits implanted with decellularized trachea survived for one year^19^. Furthermore, excellent long-term clinical results have been reported in patients, including children, by culturing stem cells or tracheal consisting cells, or adding several growth factors in the decellularized trachea^20^. However, most studies have problems such as obstruction and degradation caused by granulation and deformation due to the reduced strength in the decellularization process^21^. Three requirements for application in tracheal substitutes, (1) mechanical strength, (2) biocompatibility, and (3) nutritional vascular network, remain to be met.

Recently, there have been attempts in fabricating scaffold-free trachea-like tissues consisting only of cells and their products using a 3D bio-printer^2^. However, when scaffold-free trachea-like tissues were circumferentially implanted into the rat trachea, a combination of stents or catheters was required to maintain the lumen because of the insufficient mechanical strength^22^. A functioning capillary network is also needed in TETs to ensure the survival of the constituting cells after implantation. In fact, cultured cartilage incorporated into polycaprolactone scaffolds degrades the cartilage matrix owing to the lack of capillaries in TETs, thereby increasing the risks of infection and cell death in the cartilage tissue^5^.

In this study, a scaffold-free tracheal analogue tissue consisting of a shape-controlled cartilage ring joined by a connective tissue membrane with an in vivo generated capillary network (Fig. 2A,B) was developed. The proposed TET exhibited sufficient mechanical strength, which was equivalent to or greater than that of a native trachea (Fig. 2E). To the best of our knowledge, this is the first report that satisfies all the requirements for TETs as tracheal substitutes. Furthermore, the tracheal analogue maintained its performance without significant deformation, inflammation, or granulation formation at least 8 months after its circumferential implantation in the rat trachea (Fig. 4A-1,B); in addition, there was no degradation of the cultured cartilage rings (Fig. 4D). These results are attributed to the connection of the living and functioning capillary network in the tracheal analogues to the host blood vessels after implantation, which rapidly supplied oxygen and nutrients to the tracheal analogues, including the cartilage tissue (Fig. 3B-2). Similarly, Taniguchi et al. reported a scaffold-free trachea-like tissue fabricated from chondrocytes and vascular endothelial cells using a 3D bioprinter, which survived and formed a cartilage tissue after implantation due to the formation of a capillary network and blood flow from the host blood vessels^22^.

Although a notable survival period of 8 months was achieved using the developed tracheal analogue in rats in this study, approximately 60% of implanted rats died a day after implantation due to tracheal obstruction. This is mainly ascribed to the retention of secretions and sputum, not the granulation or inflammation at the anastomosis. In particular, small amounts of postoperative secretions can easily obstruct the trachea in rats owing to their narrow lumen, and it was technically difficult to even continuously remove secretions and sputum. Thus, we believe that in larger animal models, even if the volume of secretions and sputum increases, post-operative management similar to that of clinical patients such as endotracheal suctioning and inhaled steroid therapy would improve survival rates.

In addition to the cartilage ring, the ciliated epithelial layer of the lumen is an important tissue in the trachea, which serves as a barrier against pathogens such as viruses, bacteria, and other foreign substances. In this study, we observed the migration of the epithelial cells from the anastomosis with the native trachea to the luminal surface of the tracheal analogues 1 month after the implantation (Fig. 4A-2). Mucosal-producing goblet cells were observed in the epithelial layer 8 months after the implantation (Fig. 4C-1), indicating the maturity of the epithelial layer similar to that of the native trachea. Here, the survival of the epithelium in the allografts implanted into the trachea depends on vascular preservation and angiogenesis^23^^,^^24^. Therefore, the epithelial cell coverage of the TETs should be first induced with angiogenesis to reduce the risk of foreign body adsorption and infection until epithelialization. Thus, tracheal analogues are expected to induce a more rapid epithelial layer formation than that of conventional TETs, which have a functional capillary network generated in vivo (Fig. 3B-2).

It is essential to scale up the size of the TETs evaluated in large animals for further clinical research. However, to the best of our knowledge, there are only a few examples of scaffold-free tracheal-like tissues of the same size as that of human tissues. This is attributed to the inability to produce centimetre-scale tissues while keeping the cells alive without a functioning capillary network in the culture stage. To solve this problem, significant research needs to be conducted on the construction of culture capillary networks. In our CAT-based tissue preparation method, the size of the cultured cartilage can be easily controlled by changing the size of the culture dish. Particularly, we successfully produced cartilage rings with a diameter of more than 1.5 cm, which is equivalent to that of the trachea of a human child (Supplementary Information). Additionally, the fabrication of the iBTA tissues, tube-like tissues with diameters ranging from 0.6 to 20 mm which are applicable as artificial blood vessels^13^^,^^25^, can be easily fabricated by changing the mold size. Therefore, we believe that the proposed tracheal analogues in this study can be scaled up without losing their functionality.

There have been several attempts in generating functional tissues with a capillary network using an in vivo tissue engineering approach^26^. Cells are embedded in scaffold gels, such as collagen gels, gelatin gels, or Matrigel, and implanted in vivo to organize the cells into tissues with a capillary network. Morritt et al. reported that a spontaneously beating cardiac tissue mass with a capillary network was generated by implanting cardiomyocytes suspended in Matrigel around arteriovenous loops formed in a tissue engineering chamber in vivo^27^. However, it was difficult to control the shape of such scaffold gels based on in vivo tissue engineering techniques. In contrast, the iBTA technique has been used in generating tissues of various 3D shapes with complicated semicircular pocket structures, such as tube, sheet, and heart valve-like tissues^13–17^. In this study, the main component of the iBTA tissues is the connective tissue consisting of fibroblasts, collagen, and capillary vessels. If the tissue-specific cells could add iBTA tissues before implantation, iBTA tissue with both the desired shape and tissue-specific cell components could be obtained. We have previously demonstrated the use of cell-filled porous tubes as iBTA induction molds, which allow the cells to migrate through the pores on the sides of the molds and incorporate them into iBTA tissues encapsulated around the porous tubes^28^. However, while we could incorporate vascular component cells, such as endothelial and smooth muscle cells, into iBTA tissues, it was difficult to control the cell distribution to ensure an orderly layered structure of the endothelial and smooth muscle cells similar to the native blood vessels. In the present study, we obtained shape-controlled cartilage rings in conventional culture dishes using a tissue preparation method by CAT. By combining these cartilage rings with the iBTA induction mold and implanting them into the subcutaneous skin, we fused the cartilage rings with the collagenous tissue that forms around the mold, and a tracheal analogue structure with a regular arrangement of the cartilage rings was generated. Therefore, we believe that this method can be widely applied in creating various organs by designing the shape of the iBTA induction mold and culturing the tissue pieces prepared by CAT using tissue-specific cells or stem cells.

## Conclusion

In this study, we successfully developed a scaffold-free tracheal analogue by the in vitro preparation of cartilage rings using a cell self-aggregation-inducing culture surface and connected them with the in vivo self-assembly of connecting tissue membranes comprising a capillary network via subcutaneous embedding. The developed tracheal analogues exhibited sufficient strength and formed a capillary network similar to a native trachea. In addition, the functionality of the developed tracheal analogues was maintained without deformation 8 months after the circumferential implantation, leading to the complete replacement of the trachea with cartilage ring retention and mucosa-producing epithelial regeneration. These results indicate the possibility of easily producing scaffold-free tissues with the desired shape, strength, and a functional vascular network by combining a cell self-assembly-based in vitro tissue preparation technique with an in vivo tissue self-assembly technique.

## Methods

All animal experiments were performed under general anesthesia in accordance with the Guide for the Care and Use of Laboratory Animals, published by the United States National Institutes of Health (NIH Publication No. 85-23, revised 1996) and the ARRIVE guidelines. The research protocol was approved by the Ethics Committee of Osaka University (No: 27-050-012).

### Preparation of cartilage ring

The CAT-coated plate was prepared as follows: A cylindrical pillar (3 mm in diameter) was centered on the bottom of a 48-well plate (Thermo Fisher Scientific, MA, USA). Zwitterionic polymers synthesized for CAT^10^ were coated on the ring-shaped grooves (3.5 mm wide) of the plate via an aqueous solution (1.0 μg/cm^2^) and dried at 60 °C for 1 h.

The chondrocytes isolated from the rib cartilage of the green fluorescent protein (GFP) transgenic, or wild-type Lewis rats (Charles River Laboratories Japan, Kanagawa, Japan) were seeded on the coating surface at 4.0 × 10^5^ cells/well. The cells were incubated with 1 mL Dulbecco's modified Eagle medium (FUJIFILM Wako Pure Chemical; Osaka, Japan) with 1% penicillin streptomycin (FUJIFILM Wako Pure Chemical), 10% fetal bovine serum (Thermo Fisher Scientific, Tokyo, Japan), 50 μg/mL ascorbic acid diphosphate (FUJIFILM Wako Pure Chemical), and 10 ng/mL TGF-β1 (Pepro Tech, NJ, USA). The cartilage ring was formed after culturing for three weeks with medium changes every three days.

### Mold assembly for iBTA

The obtained cartilage rings were passed through a silicone tube (outer diameter of 3 mm) reinforced with a stainless-steel rod. The outer circumference was evenly surrounded by five stainless steel wires with a diameter of 1 mm. The distance between the silicone tube and wire was set to 1.5 mm. The mold was assembled by using a 3D printer (ProjetTM HD3000; SC, USA) after fixing the silicone tube and wires with two acrylic disks with a diameter of 9 mm.

### Preparation of tracheal analogue

The prepared molds were embedded in the dorsal subcutaneous pouches of Lewis rats (8–12-week old, 200–300 g, Charles River Laboratories Japan) under 1–2% isoflurane (Merck & Co., Inc., NJ, USA) anesthesia. The molds were harvested from the skin of the rats after four weeks. The excess tissues around them were removed. Tracheal analogues (internal diameter of 3 mm) were obtained by removing the molds, and immediately cut into 3–3.5 mm length for implantation.

### Ring compression

Compression of the analogues and native tracheas were performed using a universal tester (4464, Instron, MA, USA). A load cell (USM-5 N, Unipalse, Tokyo, Japan) was connected to a compression plate for accurate force measurements. The tracheal analogues (n = 5, outer diameter: 4.4 ± 0.2 mm, inner diameter: 3.9 ± 0.2 mm, ring width: 2.8 ± 0.3 mm) and native rat tracheas (n = 3, outer diameter: 2.9 ± 0.1 mm, inner diameter: 2.5 ± 0.1 mm, ring width: 2.9 ± 0.1 mm) were placed on the sample stage of the universal tester. The test was performed in a water bath containing a saline solution at room temperature. The contact point was defined as the place where 0.01 N was measured. The vertical deflection was calculated by multiplying the time from contact with the outer surface of the samples and the stage velocity using the universal tester. The compression was performed until the 50% strain with a constant speed (0.6 mm/min). The sampling rate was 10 Hz. The deforming curves were obtained by dividing the load value by the sample longitudinal length to compare samples of different length. The gradient of the load per unit ring width as a function of strain was used to determine the ring resistance to small deformations between 0.05 and 0.1 strain. Results were expressed as the mean ± standard deviation (SD). The t-test was used to check for significant differences among the groups, and *P* < 0.05 was considered statistically significant.

### Tracheal implantation

General anesthesia was administered to Lewis rats (12–16-weeks old, 350–430 g; Charles River Laboratories Japan) by the inhalation of 1.0–2.5% isoflurane and injection of pentobarbital (10 mg/kg intraperitoneal, Merck & Co., Inc.) An incision was made in the midline neck of the rats. The cervical trachea was exposed from the cricoid ring to the suprasternal notch and dissected from the esophagus. Three cartilage rings of the cervical tracheal segment were resected. We switched to intubation in the operative field. Anastomoses were performed at the proximal and distal ends of the tracheal analogue graft having 3 rings. The rats were allowed to recover gradually after the discontinuation of the isoflurane delivery. Analgesia (buprenorphine 0.017 mg/kg/day, subcutaneous) was administered regularly for the first five postoperative days. After the predetermined periods of implantation (1 week, 1 month, or 8 months), the rats were sacrificed by injecting sodium pentobarbital (200 mg/kg), and the implants with the surrounding tracheas were harvested.

### Histological and immunohistochemical analyses

The tracheal analogues before and after implantation were fixed in a 4.0% paraformaldehyde phosphate-buffered saline (PBS) solution (pH 7.4), dehydrated with an alcohol series, embedded in paraffin, and cut into 3–5-µm thick sections. The sections were deparaffinized, rehydrated, and stained with hematoxylin and eosin (HE), alcian blue stain or vonkossa stain. The deparaffinized sections were microwaved for 15 min at 150 W in a 0.1 M citrate buffer (pH 6.0) for α-tubulin, CD68 and vimentin antigen retrieval, and Tris–EDTA buffer (pH 9.0) for the CD31 antigen retrieval. Subsequently, the sections were washed in distilled water for 10 min, blocked with 0.1% bovine serum albumin in PBS at 20–25 °C for 1 h, and incubated with mouse anti-α-tubulin antibody (1:100, ab7291; Abcam; Cambridge, UK) and rabbit anti-CD68 antibody (1:100, ab125212; abcam), vimentin antibody (1:200, ab80667, abcam) and CD31 antibody (1:50, ab28364; Abcam) , overnight at 4 °C. After washing with distilled water for 10 min, the sections were incubated with Alexa Fluor®594 rabbit anti-mouse IgG antibody (1:1000, ab150128 abcam) or Alexa Fluor® 488 goat anti-rabbit IgG antibody (1:1000, ab150077 Abcam) at room temperature for 1 h. Subsequently, the sections were mounted using an anti-quenching reagent containing 4′,6-diamidino-2-phenylindole (DAPI) counterstain (ProLong® Gold Antifade Reagent with DAPI; Thermo Fisher Scientific) for 5 min. Fluorescent microscopy (ECLIPS Ts2; Nikon; Tokyo, Japan) was carried out to observe the sections. For the GFP-expressing cell-tracing experiments, the tracheal analogues were fixed in a 4.0% paraformaldehyde PBS solution (pH 7.4), cryo-embedded in an optimal cutting temperature compound, and cut into 20 µm-thickness at − 20 °C as frozen sections. After washing in distilled water for 20 min, the sections were observed by fluorescence microscopy.

## Supplementary Information

Supplementary Information.

## Data Availability

Data for all submitted results is available from the corresponding author on reasonable request.
