# Supplementary material for: Successful tracheal regeneration using biofabricated autologous analogues without artificial supports
Source: Sci Rep. 2022 Nov 24;12:20279. doi: 10.1038/s41598-022-24798-y (PMC9700768; doi:10.1038/s41598-022-24798-y)
Supplement: Supplementary file 1 — Supplementary Information. [file 41598_2022_24798_MOESM1_ESM.pdf]

Supplementary Information

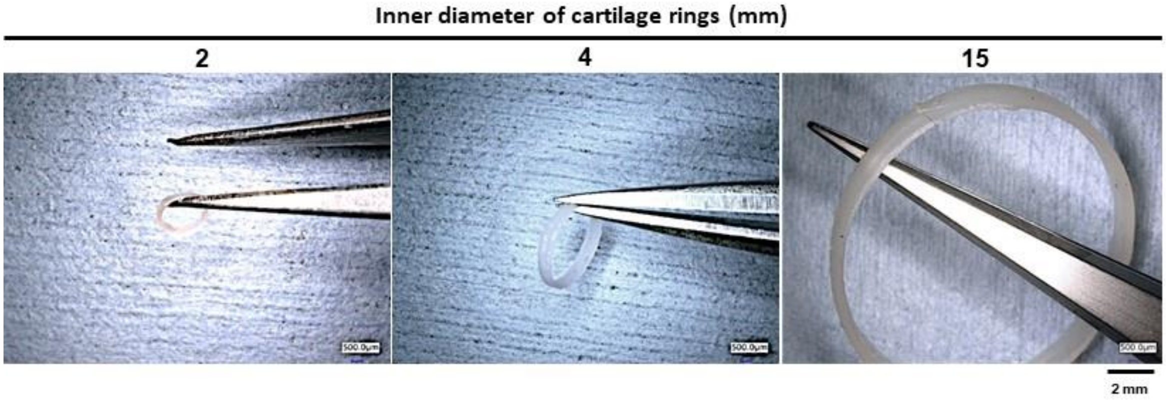

Macroscopic view of cartilage rings with different inner diameter. Mature cartilage rings with inner diameters of 2, 4, and 15 mm formed around the cylindrical pillars of 2, 4 and 15 mm in diameter, respectively, were obtained by seeding chondrocytes on CAT-coated culture grooves.
